# Supplementary material for: Highly anisotropic Fe3C microflakes constructed by solid-state phase transformation for efficient microwave absorption
Source: Nat Commun. 2024 Feb 19;15:1497. doi: 10.1038/s41467-024-45815-w (PMC10876570; doi:10.1038/s41467-024-45815-w)
Supplement: Supplementary file 1 — Supplementary Information [file 41467_2024_45815_MOESM1_ESM.pdf]

Supporting Information for

Highly Anisotropic Fe_3C Microflakes Constructed by Solid-State Phase Transformation for Efficient Microwave Absorption

Rongzhi Zhao [1,2,3], Tong Gao [1,2,3], Yixing Li [1,2,*], Zhuo Sun [2], Zhengyu Zhang [2], Lianze Ji [1], Chenglong Hu [1], Xiaolian Liu [1], Zhenhua Zhang [1], Xuefeng Zhang [1,2,*], Gaowu Qin [2]

[1] Institute of Advanced Magnetic Materials, College of Materials and Environmental Engineering, Hangzhou Dianzi University, Hangzhou 310012, China.

[2] Key Laboratory for Anisotropy and Texture of Materials (MOE), School of Materials Science and Engineering, Northeastern University, Shenyang 110819, China.

[3] These authors contributed equally to this work.

[*] Corresponding author. liyx@mail.neu.edu.cn (Y. X. Li) zhang@hdu.edu.cn (X. F. Zhang)

This file includes:

Supplementary Figures 1 to 15

Supplementary Tables 1 to 2

Supplementary References 1 to 26

Supplementary Figures

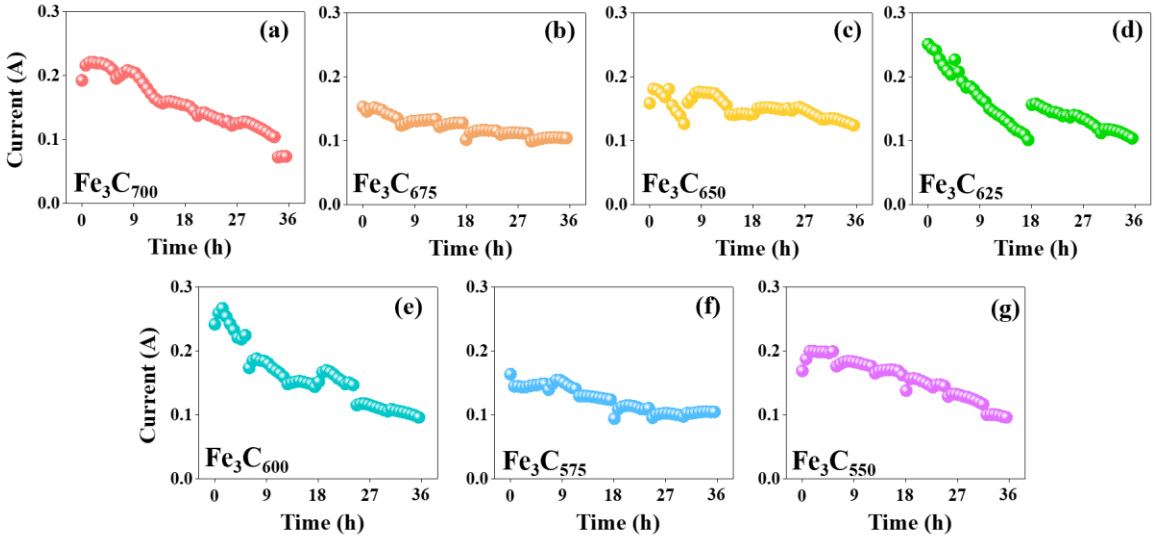

Supplementary Fig. 1. Current changing curves of all the samples during CV electrochemical dealloying process. (a) Fe_3C-700, (b) Fe_3C-675, (c) Fe_3C-650, (d) Fe_3C-625, (e) Fe_3C-600, (f) Fe_3C-575, and (g) Fe_3C-550. Source data are provided as a Source Data file.

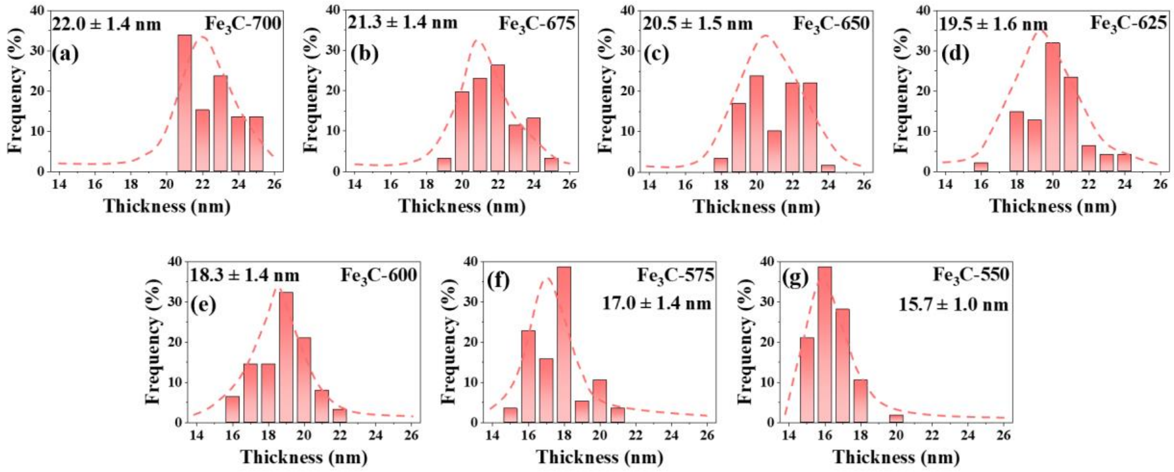

Supplementary Fig. 2. The statistic results and the fitting lines (red dashed line) of the thickness of cementite textures. (a) Fe_3C-700, (b) Fe_3C-675, (c) Fe_3C-650, (d) Fe_3C-625, (e) Fe_3C-600, (f) Fe_3C-575, and (g) Fe_3C-550. Source data are provided as a Source Data file.

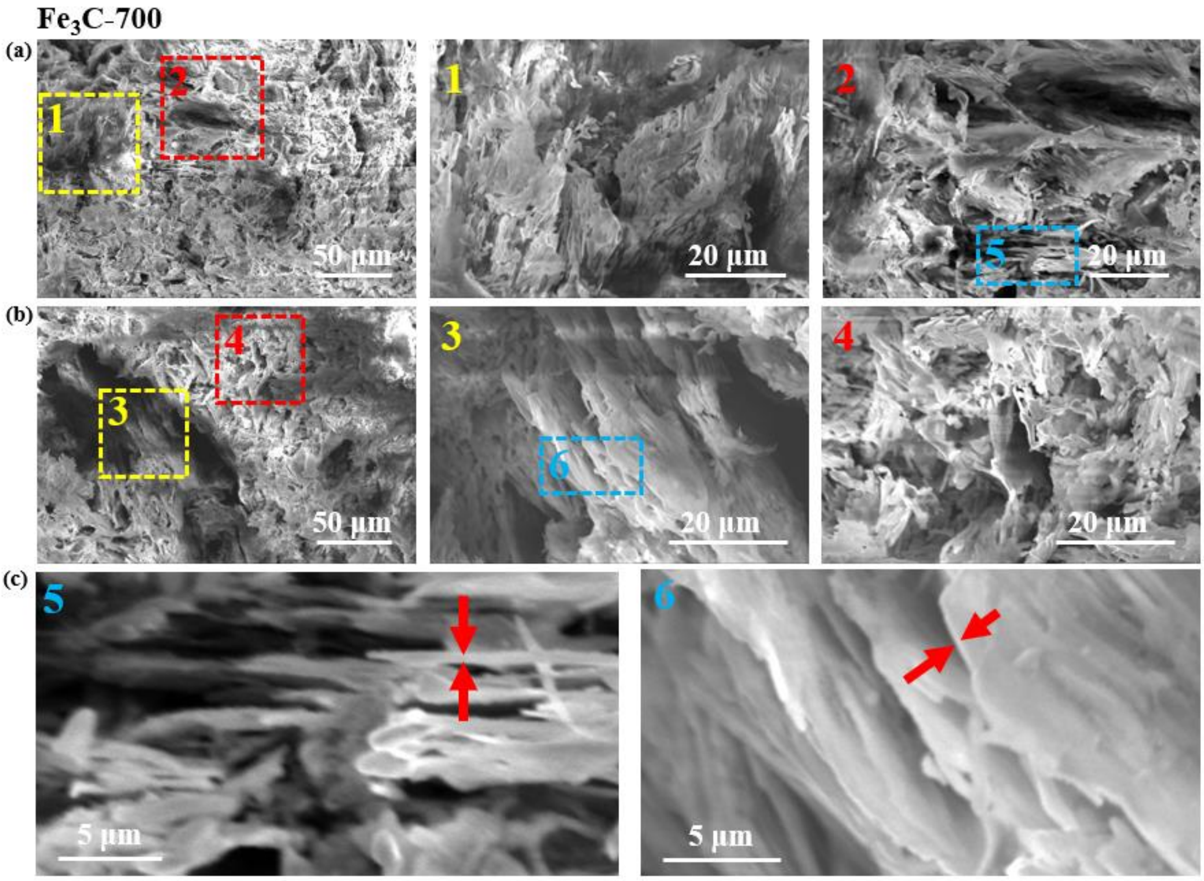

Supplementary Fig. 3. SEM images of the eutectoid steels after 48 h electrochemical dealloying process. (a) and (b) The SEM and HR-SEM images of Fe_3C after the dealloying process. (c) The HR-SEM images of the blue dashed box in (a) and (b), demonstrate the micro-flake structure of Fe_3C.

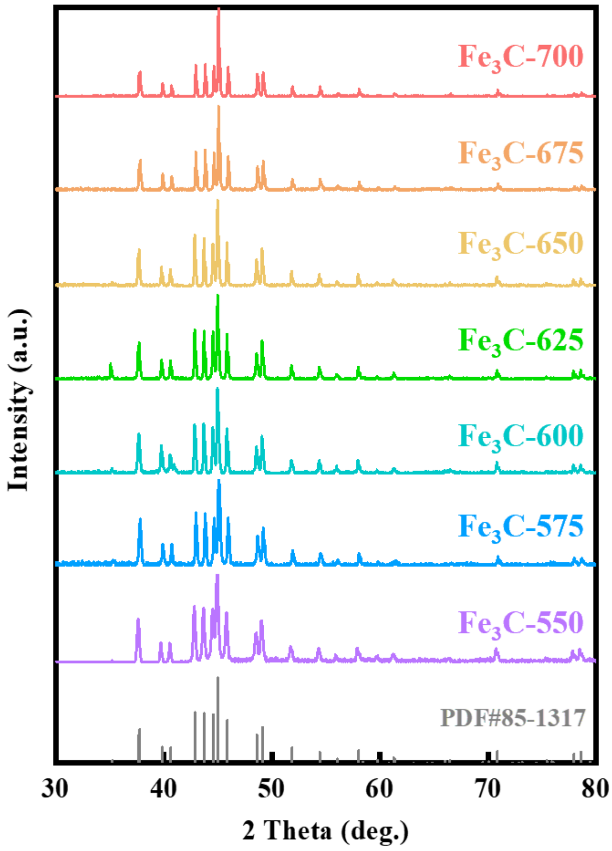

Supplementary Fig. 4. XRD patterns of the different Fe_3C micro-flakes. Source data are provided as a Source Data file.

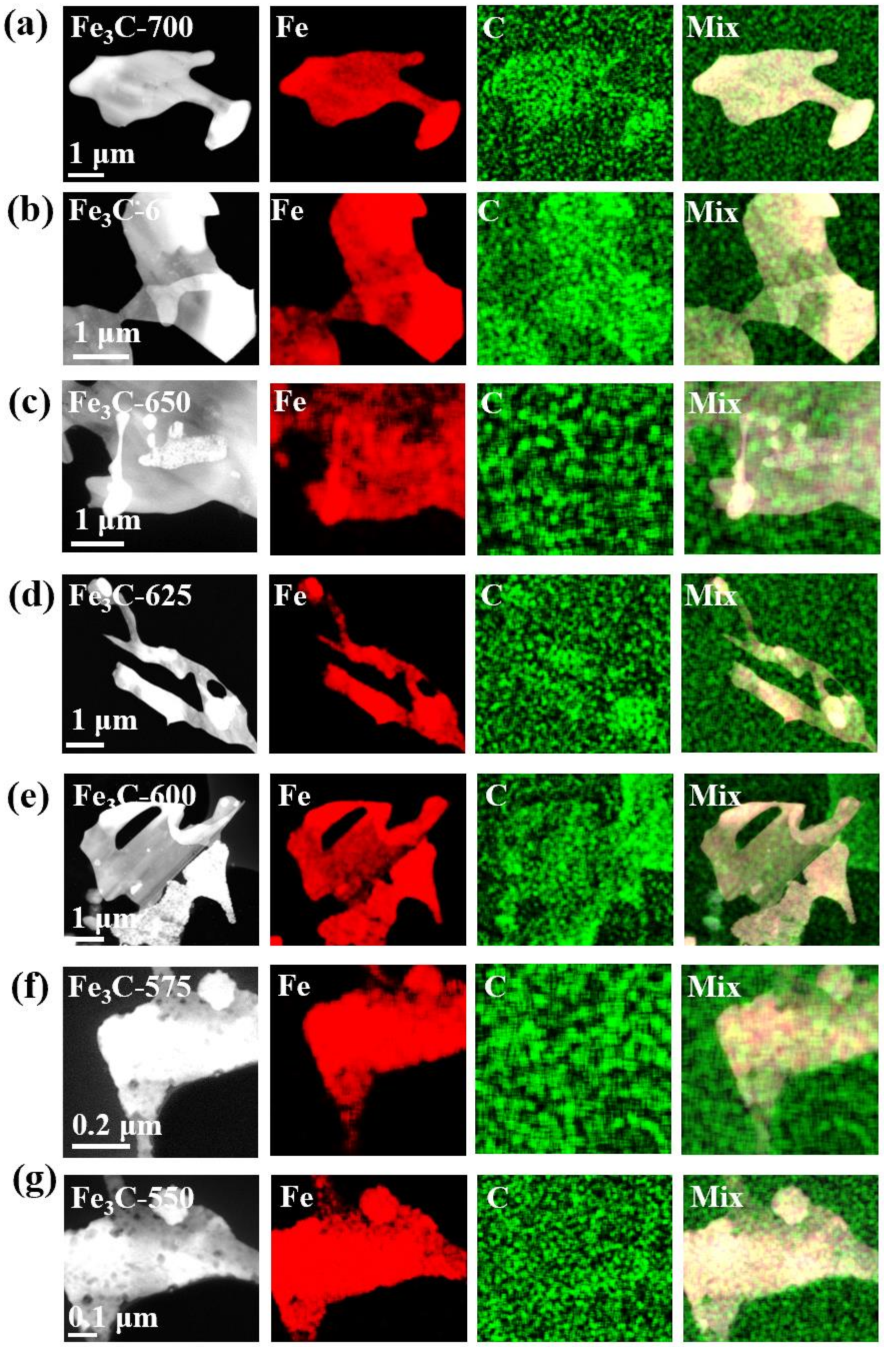

Supplementary Fig. 5. Dark field TEM images and the corresponding EDS mapping results. (a) Fe_3C-700, (b) Fe_3C-675, (c) Fe_3C-650, (d) Fe_3C-625, (e) Fe_3C-600, (f) Fe_3C-575, and (g) Fe_3C-550.

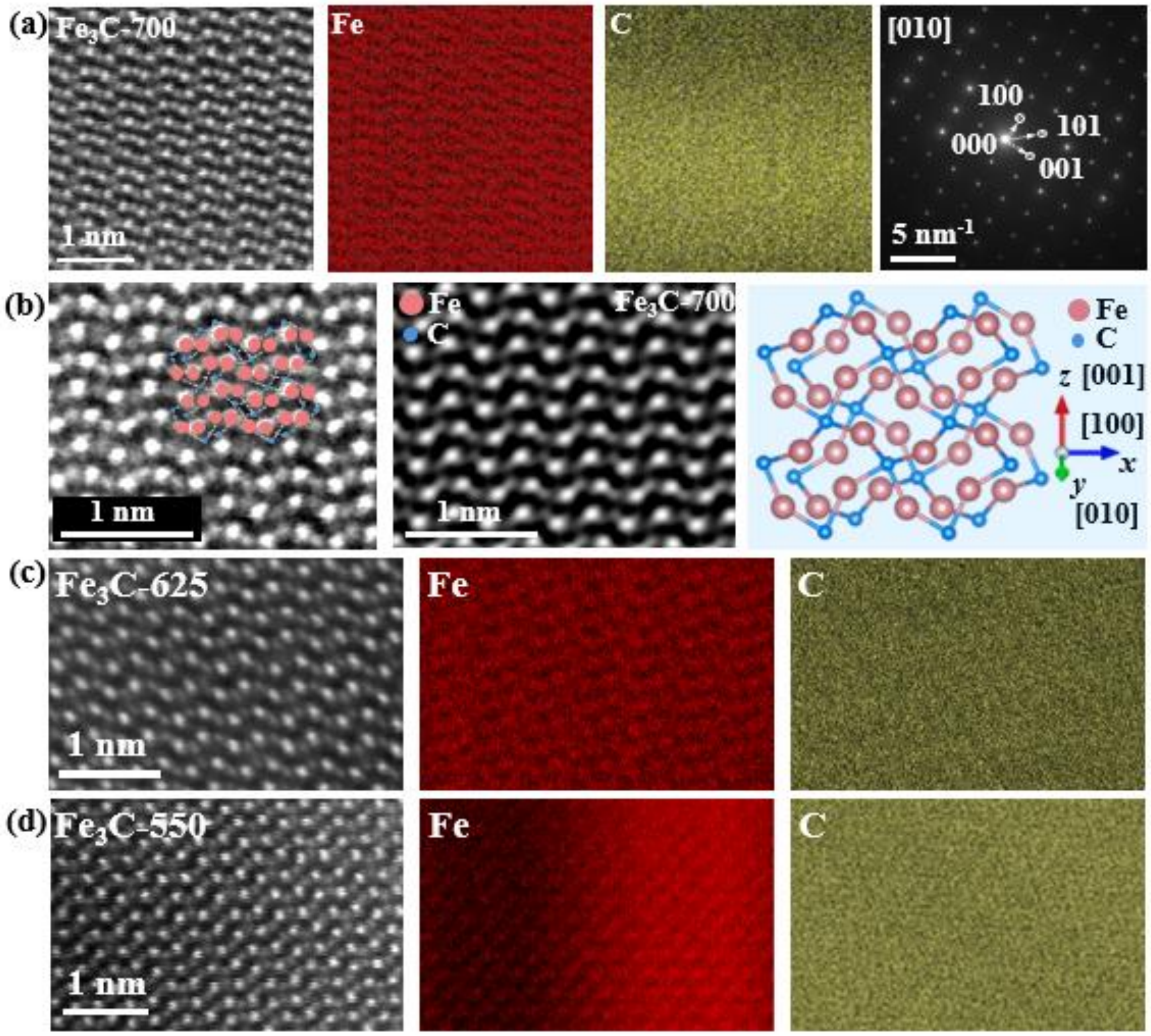

Supplementary Fig. 6. Misstructure characterizations. (a) High-resolution TEM image, atomic EDS maps, and SAED image of Fe3C-700 microflakes. (b) Magnified HR-TEM and the corresponding IFFT images of Fe3C-700 microflakes and the crystalline structure. And high-resolution TEM images and the corresponding atomic EDS maps of (c) Fe_3C-625 and (d) Fe_3C-550 micro-flakes.

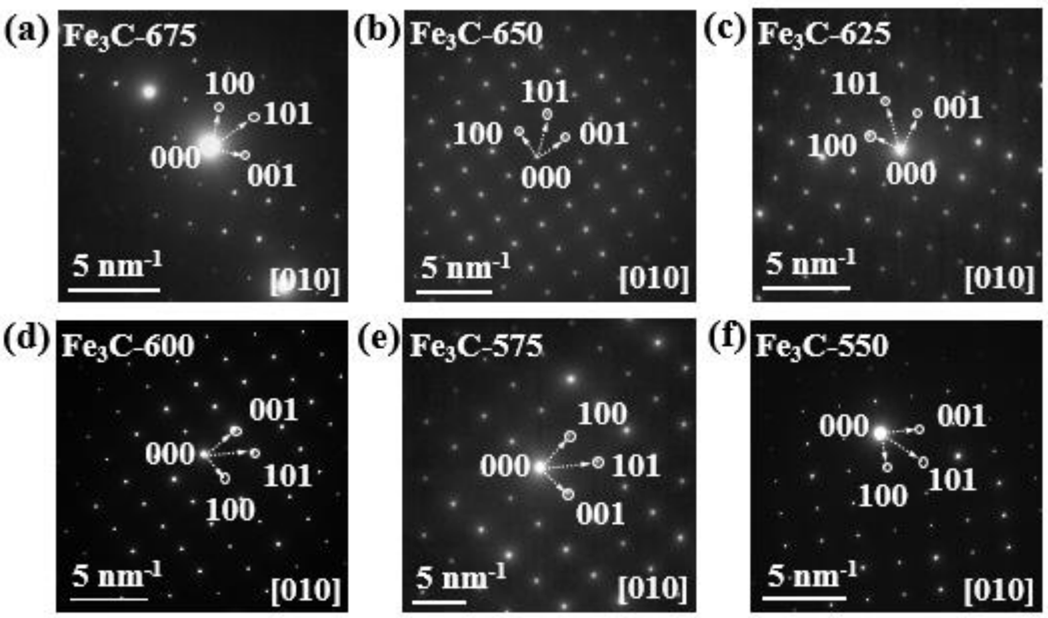

Supplementary Fig. 7. SAED images of Fe_3C micro-flakes. (a) Fe_3C-675, (b) Fe_3C-650, (c) Fe_3C-625, (d) Fe_3C-600, (e) Fe_3C-575, and (f) Fe_3C-550.

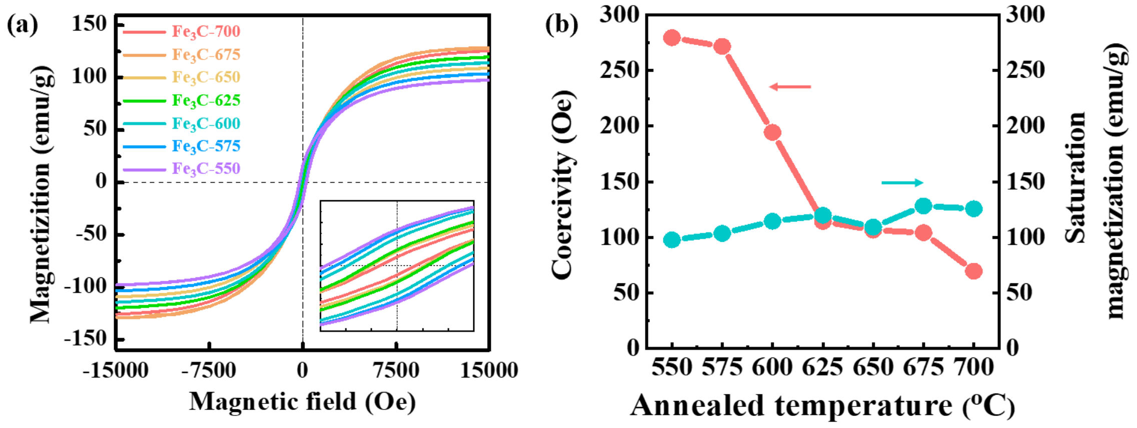

Supplementary Fig. 8. Magnetic properties. (a) Hysteresis loops of the Fe_3C micro-flakes and (b) the summarizations of coercivity and saturation magnetization. Source data are provided as a Source Data file.

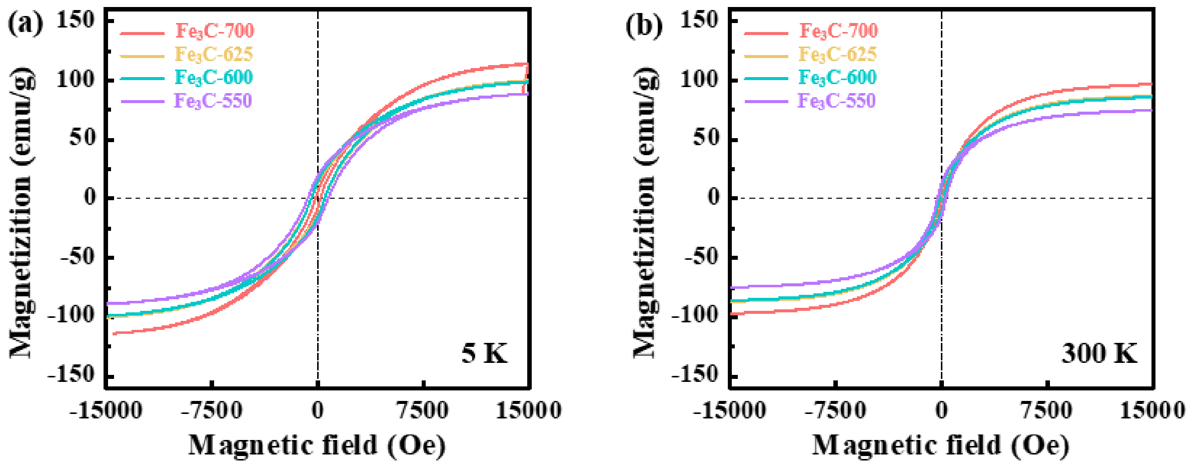

Supplementary Fig. 9. Magnetic properties at different temperature. Hysteresis loops of the Fe_3C micro-flakes at (a) 5 K, and (b) 300 K. Source data are provided as a Source Data file.

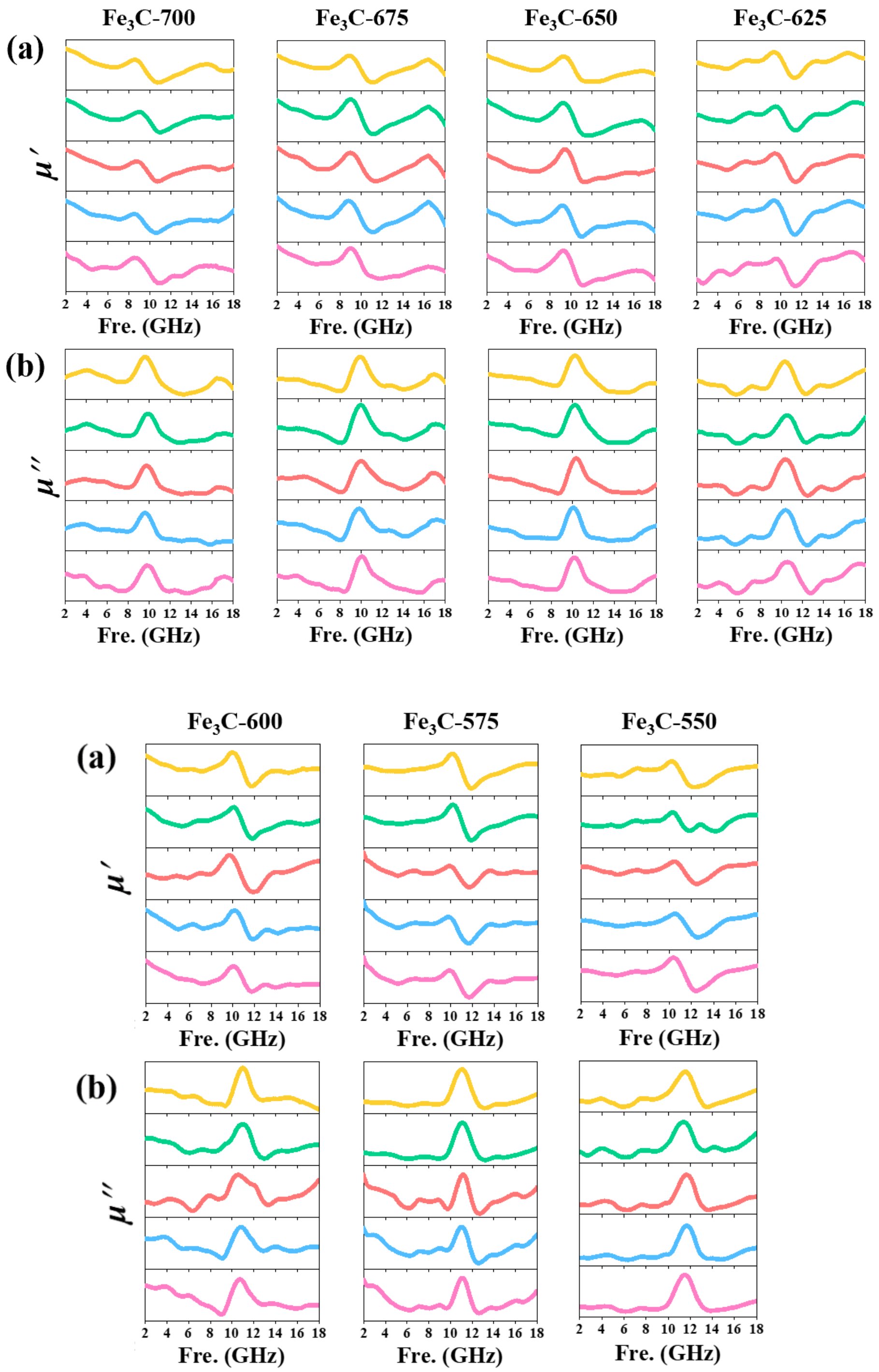

Supplementary Fig. 10. Magnetic abilities. (a) Real part and (b) imaginary part of complex permeability of all the Fe_3C micro-flakes. Source data are provided as a Source Data file.

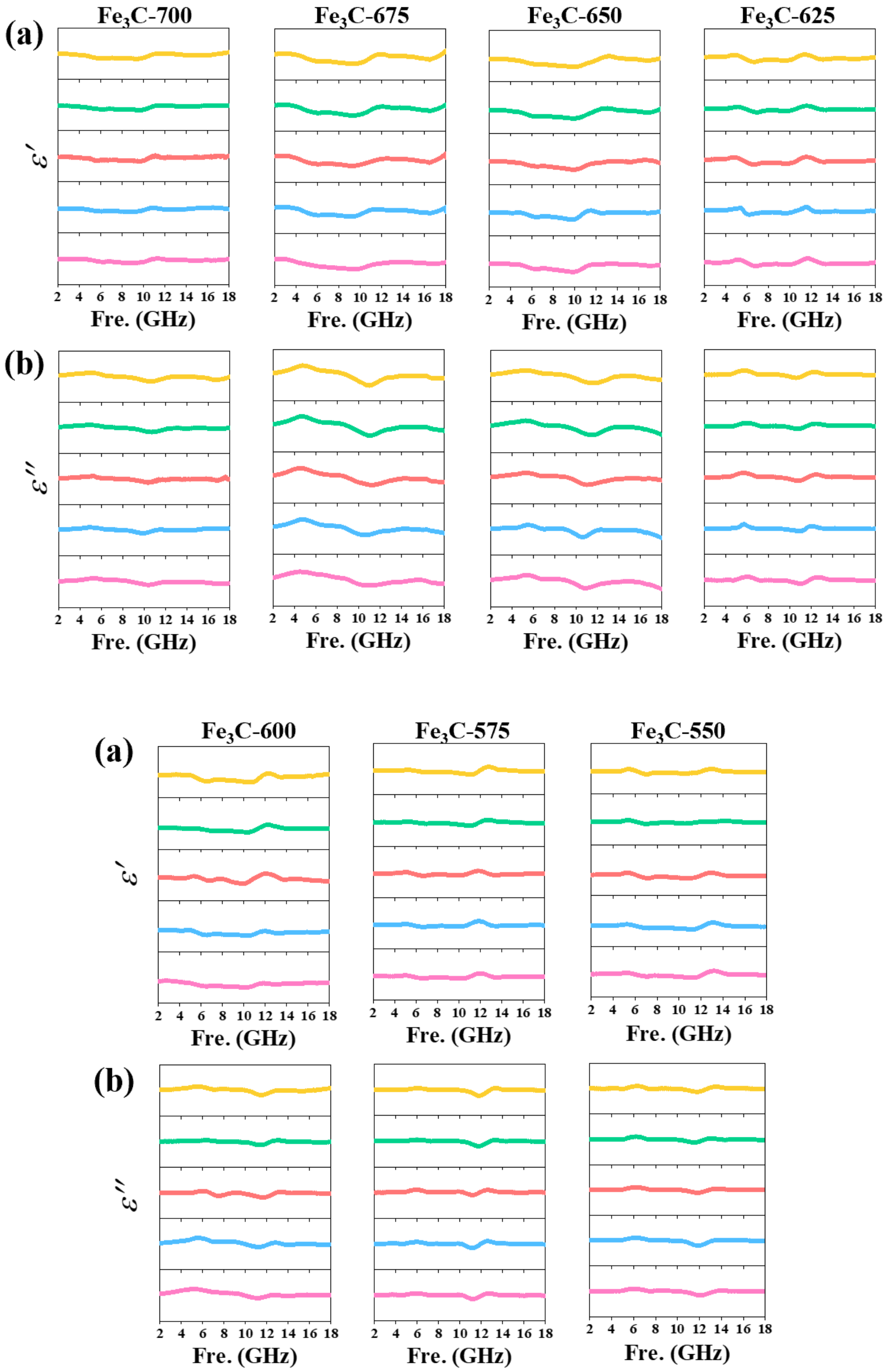

Supplementary Fig. 11. Dielectric abilities. (a) Real part and (b) imaginary part of complex permittivity of all the Fe_3C micro-flakes. Source data are provided as a Source Data file.

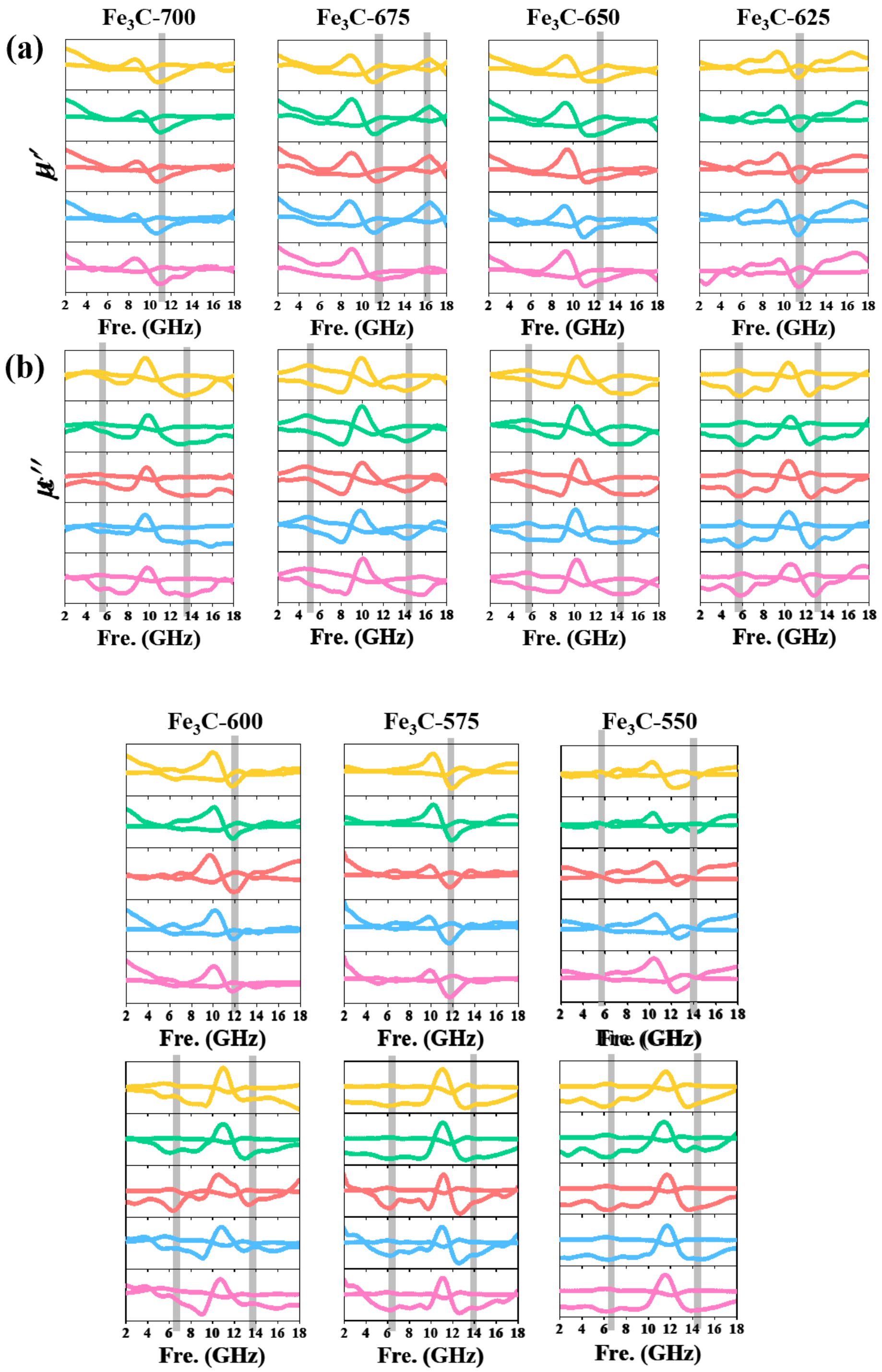

Supplementary Fig. 12. The superimposed figure of complex permittivity (Supplementary Fig. 11) and complex permeability (Supplementary Fig. 10) of all five experimental results has been used in the manuscript, in which the corresponding resonance position can be noticed. (a) Real part and (b) imaginary part.

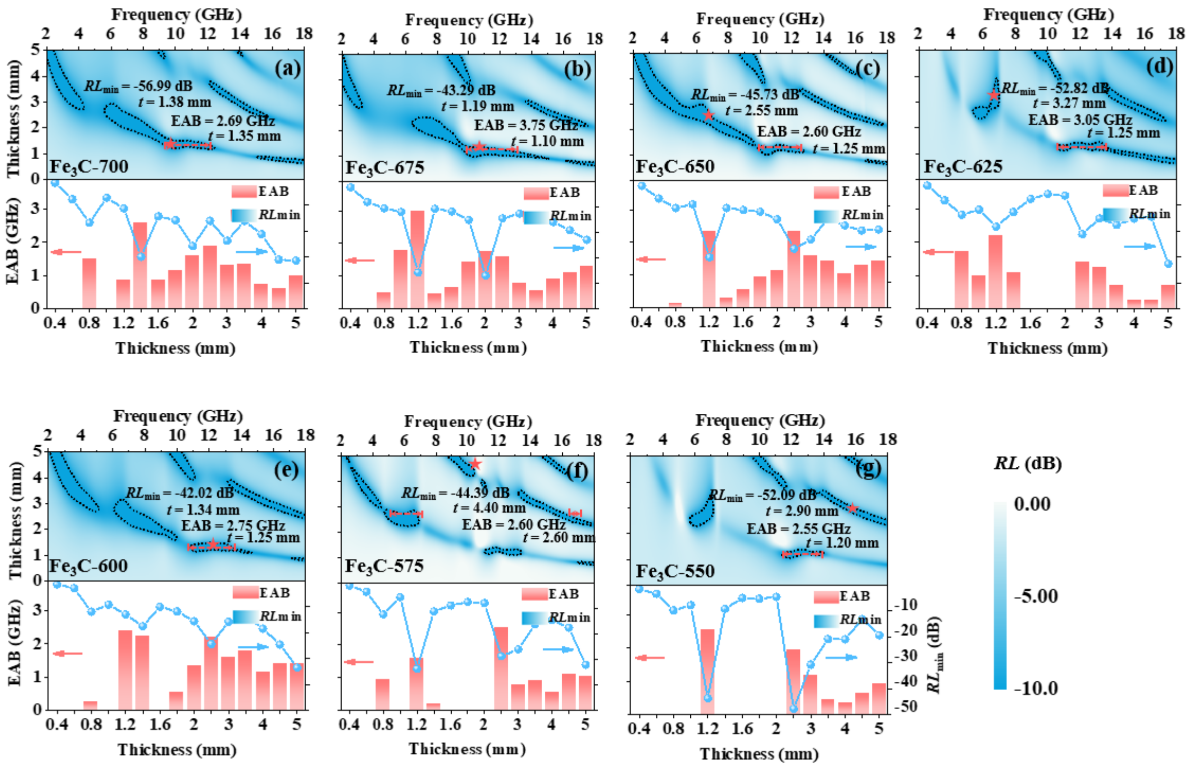

Supplementary Fig. 13. 2D *RL* maps and the corresponding EAB summarizations of Fe_3C micro-flakes. (a) Fe_3C-700, (b) Fe_3C-675, (c) Fe_3C-650, (d) Fe_3C-625, (e) Fe_3C-600, (f) Fe_3C-575, and (g) Fe_3C-550. Source data are provided as a Source Data file.

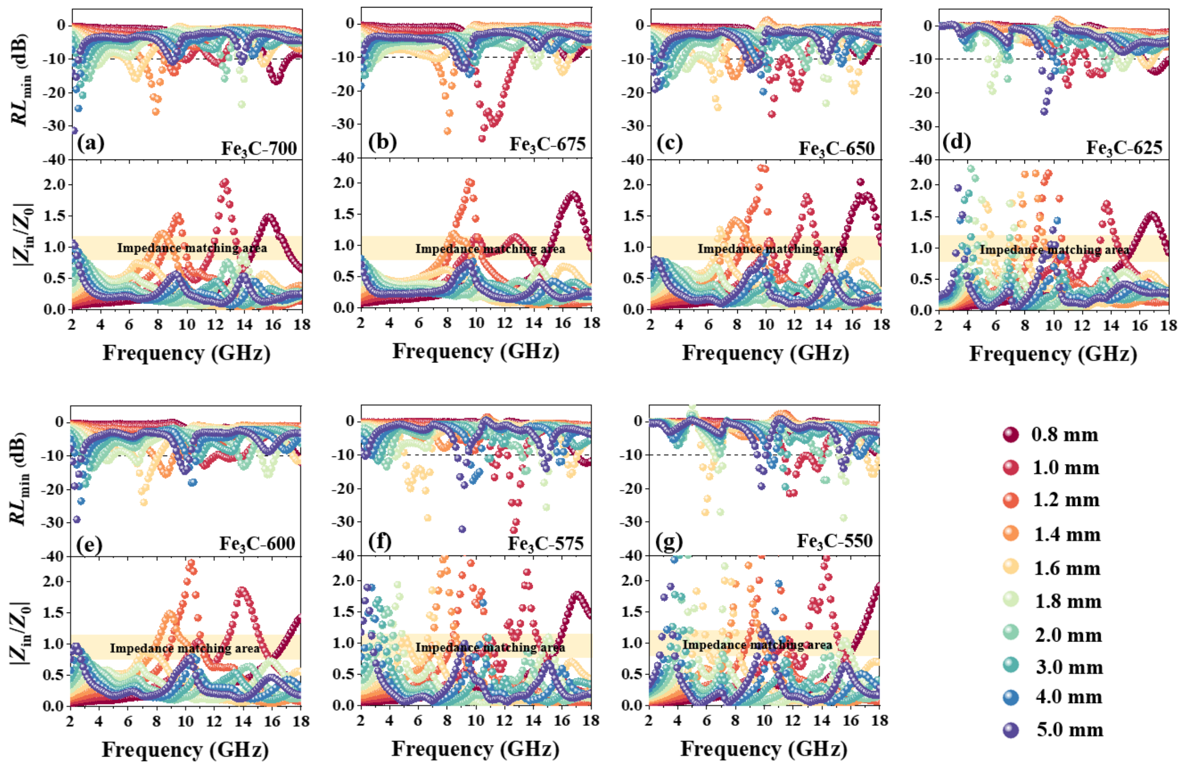

Supplementary Fig. 14. *RL_{min}* and $|Z_{in}/Z_0|$ values (impedance matching) of Fe_3C micro-flakes at the selected thickness. (a) Fe_3C-700, (b) Fe_3C-675, (c) Fe_3C-650, (d) Fe_3C-625, (e) Fe_3C-600, (f) Fe_3C-575, and (g) Fe_3C-550. Source data are provided as a Source Data file.

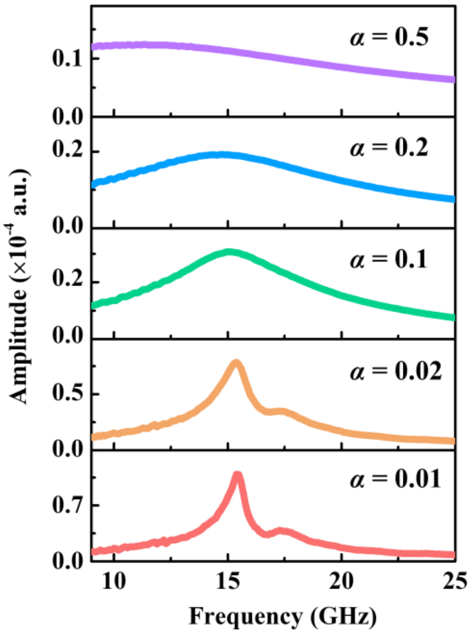

Supplementary Fig. 15. The influence of damping constant on dynamics. Because the damping constant can affect the amplitude of resonant peaks, some other spin wave modes can be found under smaller damping constants (0.01 and 0.02) and the main spin wave modes can be hid under larger damping constants (0.5). Source data are provided as a Source Data file.

Supplementary Table 1. The fitted damping constant of all the samples.

Samples	Fe_3C-550	Fe_3C-575	Fe_3C-600	Fe_3C-625	Fe_3C-650	Fe_3C-675	Fe_3C-700	Average
α	0.073	0.056	0.090	0.078	0.107	0.134	0.140	0.097

Supplementary Table 2. The experimental and simulated amplitude values of imaginary part of complex permeability.

Samples	Experimental values	Simulated values($\times10^{-4}$)
**Fe_3C-550**	0.91	0.28
**Fe_3C-575**	0.97	NaN
**Fe_3C-600**	0.84	0.29
**Fe_3C-625**	0.86	0.13
**Fe_3C-650**	1.47	0.14
**Fe_3C-675**	1.26	NaN
**Fe_3C-700**	1.07	0.30

Supplementary References

[1] R. Han, H. Yi, J. Wei, L. Qiao, T. Wang, F. Li, *Appl. Phys. A* **2012**, 108, 665.

[2] W. Tian, X. Zhang, Y. Guo, C. Mu, P. Zhou, L. Yin, L. Zhang, L. Zhang, H. Lu, X. Jian, L. Deng, *Carbon* **2021**, 173, 185.

[3] Y. Lei, Z. Yao, S. Li, J. Zhou, A. A. Haidry, P. Liu, *Ceram. Int.* **2020**, 46, 10006.

[4] C. Lei, Y. Du, *J. Alloys Compd.* **2020**, 822, 153674.

[5] Y. Qing, J. Su, Q. Wen, F. Luo, D. Zhu, W. Zhou, *J. Alloys Compd.* **2015**, 651, 259.

[6] J. Sun, H. Xu, Y. Shen, H. Bi, W. Liang, R.-B. Yang, *J. Alloys Compd.* **2013**, 548, 18.

[7] Y. Xu, L. Yuan, X. Wang, D. Zhang, *J. Alloys Compd.* **2016**, 676, 251.

[8] C. Guo, Z. Yang, S. Shen, J. Liang, G. Xu, *J. Magn. Magn. Mater.* **2018**, 454, 32.

[9] R. Han, H. Yi, W. Zuo, T. Wang, L. Qiao, F. Li, *J. Magn. Magn. Mater.* **2012**, 324, 2488.

[10] O. Khani, M. Z. Shoushtari, K. Ackland, P. Stamenov, *J. Magn. Magn. Mater.* **2017**, 428, 28.

[11] A. Poorbafrani, E. Kiani, *J. Magn. Magn. Mater.* **2016**, 416, 10.

[12] H. Xu, S. Bie, J. Jiang, W. Yuan, Q. Chen, Y. Xu, *J. Magn. Magn. Mater.* **2016**, 401, 567.

[13] Y. Yuan, S. Wei, Y. Liang, Y. Wang, B. Wang, W. Huang, W. Xin, X. Wang, *J. Magn. Magn. Mater.* **2020**, 506, 166791.

[14] Y. Zhang, C. Liu, X. Zhao, M. Yao, X. Miao, F. Xu, *J. Magn. Magn. Mater.* **2020**, 494, 165828.

[15] Y. Pan, G. Ma, X. Liu, C. Wang, N. Li, J. Wang, X. Jian, *J. Mater. Sci. Mater. Electron.* **2019**, 30, 18123.

[16] X. Chi, H. Yi, W. Zuo, L. Qiao, T. Wang, F. Li, *J. Phys. D: Appl. Phys.* **2011**, 44, 295001.

[17] Q. Hu, G. Qiao, W. Yang, Z. Liu, P. Zhang, S. Liu, C. Wang, D. Zhou, R. Han, D. Cai, B. Hu, J. Yang, *J. Phys. D: Appl. Phys.* **2020**, 53, 115001.

[18] C. He, S. Pan, L. Cheng, X. Liu, Y. Wu, *J. Rare Earths* **2015**, 33, 271.

[19] W. Yang, Q. Hu, G. Qiao, L. Zha, S. Liu, J. Han, H. Du, Y. Zhang, Y. Yang, C. Wang, *J. Rare Earths* **2019**, 37, 1102.

[20] K.-P. Jeong, S.-W. Yang, J.-H. Choi, J.-G. Kim, *Met. Mater. Int.* **2020**, DOI: 10.1007/s12540-020-00613-z.

[21] H. Xing, Y. Liu, Z. Liu, H. Wang, H. Jia, *Nano* **2018**, 13, 1850105.

[22] G. Tan, Y. Zhang, L. Qiao, T. Wang, J. Wang, F. Li, *Phys. B Condens. Matter* **2015**, 477, 52.

[23] Z. Zhang, J. Wei, W. Yang, L. Qiao, T. Wang, F. Li, *Phys. B Condens. Matter* **2011**, 406, 3896.

[24] W. Zuo, L. Ying, L. Qiao, T. Wang, F. Li, *Phys. B Condens. Matter* **2010**, 405, 4397.

[25] X. Song, H. Yan, Y. Wang, Z. Ma, B. Xu, *Pramana* **2018**, 91, 85.

[26] K. Jagatheesan, A. Ramasamy, A. Das, A. Basu, *Smart Mater. Struct.* **2018**, 27, 025004.
